# Evaluation of Living Donor Liver Transplantation in Germany: Opportunities for Expanding the Donor Pool

**DOI:** 10.1055/a-2769-4047

**Published:** 2026-04-22

**Authors:** Oliver Rohland, Felix Dondorf, Laura Schwenk, Aladdin Ali-Deeb, Michael Ardelt, Christina Malessa, Utz Settmacher, Falk Rauchfuß

**Affiliations:** 1 39065 Department of General, Visceral and Vascular Surgery, Jena University Hospital, Jena, Germany; 2 Interdisciplinary Center for Clinical Research (IZKF), Jena University Hospital, Jena, Germany

**Keywords:** living donor liver transplantation, donor evaluation, donor selection criteria, liver transplantation, deceased donor liver transplantation, Leberlebendspende, Donor Evaluation, Lebertransplantation, Spenderselektionskriterien, Organspende

## Abstract

**Background:**

Living donor liver transplantation (LDLT) represents an important alternative to deceased donor transplantation, particularly in times of organ shortage. This study analyzes the selection process of living liver donors at a German transplant center that performs both living and deceased donor liver transplantations.

**Methods:**

In a retrospective analysis, potential living liver donors were evaluated. Medical data and reasons for donor exclusion were collected, and recipient cohorts were categorized according to transplantation indication and urgency. Donor outcomes were also assessed.

**Results:**

Between January 2013 and December 2023, a total of 594 potential living donors were evaluated at Jena University Hospital. Of these, 124 successfully proceeded to donation. During the outpatient evaluation phase, 357 donors were excluded. Subsequently, 237 individuals underwent inpatient assessments, of whom an additional 100 were excluded. The main reasons for donor exclusion were medical or psychological contraindications (n = 160), as well as donor-independent factors such as disease progression in the recipient (n = 90) or withdrawal of consent by either the recipient or the donor (n = 54). Particularly among recipients with malignant diseases who had little or no chance of receiving a deceased donor organ, a high willingness to donate was observed—even among distant relatives or unrelated donors.

**Conclusion:**

A standardized evaluation protocol improves the efficiency of living donor liver transplantation within the Eurotransplant region and ensures donor safety. Diverse contraindications necessitate targeted exclusion diagnostics for individual potential donors.

## Zusammenfassung

**Hintergrund:**

Die Leberlebendspende bietet besonders in Zeiten des Organspendemangels eine subsidiäre Alternative zur postmortalen Leberspende. Diese Studie untersucht den Auswahlprozess von Lebendleberspendern an einem deutschen Transplantationszentrum, das beide Spendeformen anwendet.

**Methodik:**

Im Rahmen einer retrospektiven Analyse wurden potenzielle Lebendspender untersucht, medizinische Daten sowie Ablehnungsgründe für die Lebendspende erhoben und die Patientenkohorten nach Transplantationsindikation und -dringlichkeit unterteilt. Außerdem wurde das Outcome der Spender betrachtet.

**Ergebnisse:**

Zwischen Januar 2013 und Dezember 2023 wurden am Universitätsklinikum Jena insgesamt 594 potenzielle Lebendspender evaluiert. Davon konnten 124 erfolgreich spenden. Bereits während der ambulanten Evaluierung wurden 357 Spender ausgeschlossen. Im Anschluss durchliefen 237 Personen Untersuchungen unter stationären Bedingungen, von denen weitere 100 ausgeschlossen wurden. Die Hauptgründe für eine Ablehnung als Spender waren medizinische oder psychologische Kontraindikationen (n = 160), aber auch spenderunabhängige Faktoren wie das Fortschreiten der Erkrankung beim Empfänger (n = 90) sowie eine Ablehnung durch den Empfänger oder ein Rückzug der Spendebereitschaft des Spenders (n = 54). Besonders bei Patienten mit malignen Erkrankungen, welche keine oder wenig Chancen auf eine postmortale Spende hatten, zeigte sich eine hohe Spendebereitschaft auch von entfernten Verwandten oder nicht-verwandten Spendern.

**Schlussfolgerung:**

Ein standardisiertes Evaluationsprotokoll verbessert die Effizienz von Lebendleberspenden im Eurotransplant-Raum und dient der Spendersicherheit. Unterschiedliche Kontraindikationen erfordern die gezielte Ausschlussdiagnostik einzelner potenzieller Spender.

## Introduction

Liver transplantation (LT) is the standard treatment for end-stage liver disease. The shortage of deceased donor organs in Western countries has led to an extended waiting time for transplantation 1 . In recent years, donor organ quality has worsened because of organ scarcity and because most donors are elderly and have experienced a history of illness 2 3 . More than 200 patients per year on the waiting list have died in Germany in recent years 4 .

Living-donor liver transplantation (LDLT) represents a valuable complementary option to deceased-donor liver transplantation, particularly in the context of ongoing organ shortages. While not suitable for all patients, LDLT can be considered in selected cases across various indications, offering an alternative pathway to transplantation when deceased donor organs are not readily available 5 6 7 . While LDLT is the first-choice procedure in Asia, often because of religious and cultural backgrounds 8 , the proportion of living donations from all liver transplants performed in Germany is 5–7.5% 9 . Here, LDLT was established for paediatric patients 10 . Only a few centres offer programs for adult recipients 10 . Eleven centres have offered living liver donations for adults over the years. Of these, only five centres achieved case numbers of >3 LDLT/year (Jena, Hamburg, Essen, Hanover, and Regensburg) 11 .

LDLT may offer certain logistical and clinical benefits, including the potential to reduce waiting times and improve graft quality because of shorter cold ischaemia time. Moreover, the ability to plan the procedure in alignment with recipient-specific therapeutic strategies — such as neoadjuvant treatment in hepatocellular carcinoma (HCC) or managing clinical deterioration in conditions such as ongoing cholangitis in primary sclerosing cholangitis — can be advantageous 10 .

Donor safety remains a key aspect of every LDLT program 5 12 13 14 15 . A well-known template for an evaluation program was published by Trotter et al. in 2000, who reported on experiences with donor evaluation 16 . The population of 100 potential living donors at an American transplant centre was examined for exclusion criteria and contraindications to donation. An efficient but standardized psychological and medical donor evaluation is crucial.

The aim of this study is to present our evaluation process and strategy. We discuss the aspects and recommendations from the literature 15 17 and compare our program with preexisting schemes.

## Methods

This study retrospectively analysed all potential living donors assessed for LDLT at Jena University Hospital from January 2013 to December 2023. We retrospectively identified eligible patients by identifying all patients who were listed for liver transplantation, who died on the liver waiting list, and who received liver transplantation. The associated cases for potential living liver donations and the respective potential donors were subsequently extracted by querying the code for living liver donors according to the ICD-10 classification (Z52.6) for presentation in our outpatient consultation or inpatient hospital stay.

The donors were retrospectively interviewed about their experiences and the consequences of living liver donation to obtain outcome data, with a focus on medical follow-up information and potential complications, including psychological distress.

General epidemiological data on potential recipients were collected to determine whether the donor selection process differed depending on the recipient's primary diagnosis and disease severity ( Table 1 ). Therefore, we differentiated between benign and malignant underlying diseases after decompensation of liver cirrhosis. These data were collected from the digital hospital files in Sap (Sap Deutschland SE Co. KG; Walldorf, Germany) and with Copra (Copra System GmbH, Berlin, Germany).

**Table:** **Table 1** Patient characteristics. Continuous variables are presented as the mean (SD; range) and were compared between accepted and nonaccepted donors using Student’s t test. Categorical variables are presented as numbers and percentages and were compared using chi-squared test or Fisher’s exact test, as appropriate.

Characteristic		overall potential Donors (n = 594)	Donors who were Accepted (n = 124)	Donors Who Were Not Accepted (n = 470)	Accepted vs. not accepted Donors
		mean (SD; range) or n (%)	mean (SD; range) or n (%)	mean (SD; range) or n (%)	*p* value
donor	Age in years	43.67 (12.5; 18–76)	43.75 (12; 21–66)	43.65 (12.6; 18–76)	0.17
	Sex	309 Women (52%) 285 Men (48%)	73 Women (58.9%) 51 Men (41.1%)	236 Women (50.2%) 234 Men (49.8%)	0.09
	Biologically related	336 (56.6%)	80 (64.5%)	256 (54.5%)	0.04
	Relatedness grade 1	216 (36.4%)	54 (43.5%)	162 (34.5%)	0.06
	Relatedness grade 2	120 (20.2%)	26 (21%)	94 (20%)	0.81
	Not biologically related	258 (43.4%)	44 (35.5%)	214 (45.5%)	0.03
	Relatedness grade 3	141 (23.7%)	31 (25%)	110 (23.4%)	0.75
	Relatedness grade 4	117 (19.7%)	13 (10.5%)	104 (22.1%)	<0.01
recipient	labMELD	14.73	11.95	15.45	<0.01
	Malignom*	320 (53.9%)	64 (51.6%)	256 (54.5%)	0.82
	Benign	274 (46.1%)	60 (48.4%)	214 (45.5%)	0.54
	HCC inside Milan criteria	21 (16.2%)	7 (22.6%)	14 (14.1%)	0.15
	HCC outside Milan criteria	109 (83.8%)	24 (77.4%)	85 (85.9%)	0.75
	*CPT* , Child–Pugh–Turcotte Score for cirrhosis mortality; labMELD. * Malignom – hepatocellular carcinoma, intrahepatic cholangiocarcinoma, perihilar cholangiocarcinoma and colorectal liver metastases.				

Our programʼs policy entails conducting evaluations following a standardized process, in which patients progress through three levels of assessment ( Table 2 ).

**Table:** **Table 2** Evaluation Phases and Exclusion Process.

Evaluation Phase	Donor Evaluation and Rejection Criteria	Recipient Evaluation and Requirements for LDLT
*Outpatient Presentation*	Missing Donor-Recipient Relationship Previous significant abdominal surgery (especially in the upper abdomen or with significant intra-abdominal adhesions) Severe medical problems and comorbidity cardiovascular disease pulmonary disease diabetes active infection Medication Laboratory diagnostics ABO-incompatible blood group hepatitis liver dysfunction alcohol or illicit substance abuse sonographic liver fibrosis or cirrhosis and masses	Indication for liver transplantation Underlying disease underrepresented in the MELD system (HCC outside the Milan criteria, primary sclerosing cholangitis without exceptional points, adenomatosis of the liver) Liver transplantation for colorectal liver metastases in the Liver-t(w)o-Heal study LDLT as a secondary concept to expand the pool of donor organs
*Inpatient Presentation*	** imaging procedures** CT of thorax/abdomen/pelvis with contrast agent and MRCP: 3D reconstruction of the liver for volumetry, planning the resection level and visualization of the vascular anatomy* ** cardiopulmonary diagnostics** echocardiography, lung function testing using Spirometry and body plethysmography, stress ECG Other tests or consultations to clarify potential problems uncovered during evaluation or further diagnostics in case of conspicuous examination results (endoscopic retrograde cholangiography, heart catheter examination, myocardial scintigraphy, coronary CT) **operation enlightenment** (together with donor, recipient and witnesses) ** liver function test** Limax (Humedics GmbH, Berlin, Germany) **psychological and psychiatric evaluation** to exclude underlying psychiatric illness and to ensure compliance **ethics committee** **	Listing for deceased donor liver transplantation (absence of significant contraindications) according to the guidelines of the German Federal Medical Association for liver transplantation no previous psychiatric illnesses, given compliance Approval by the interdisciplinary transplantation conference (immunosuppression and plasmapheresis for ABO-incompatible living donation) *LDLT as an individual therapy concept when the option of deceased donor liver transplantation is missing*
*Operation*	Surgical exploration of the donor (Comparison of the anatomical relations and intraoperative Cholangiography)	Surgical exploration of the recipient (Check of feasibility) →recipient first for malignant diseases
* during outpatient presentation if viable ** according to the German organ donation law		

### Outpatient Consultation

Initially, the patients were referred to our outpatient department. Potential donors must clarify their voluntary living liver donation and prove their close relationship with the organ recipient. Following these steps, a survey of the detailed anamnesis and medical history was conducted, including current medication, medication history, family history, allergies, previous illnesses, and previous operations. The acceptance of blood transfusions and coagulation preparations should also be investigated. Potential donors were also physically examined. Laboratory tests included the following: blood group, transaminases, cholestasis parameters, coagulation, retention parameters, serum electrolytes, albumin, virological status, inflammation, and blood counts 18 . Abdominal sonographic examination was performed to visualize the liver parenchyma, bile ducts, and liver vessels to estimate the organ size and degree of steatosis, and to rule out malignancies.

### Inpatient Examination

The second step took place after inpatient admission. Repeated psychological interviews constitute another requirement in the evaluation process. Computed tomography (CT) and magnetic resonance cholangiopancreatography (MRCP) were performed 19 20 21 . We used 3D reconstruction software 22 to display the liver anatomy to plan the resection level, perform volumetry of the split liver, and identify anatomical variants (Mevis Medical Solutions AG; Bremen; Germany/Synapse 3D, Fujifilm; Minato, Japan) 22 . We used a noninvasive test to assess the donor’s liver function (Limax; Humedics GmbH, Berlin, Germany) 23 . The cardiopulmonary suitability of the donors was also assessed. If needed, additional examinations were performed and analysed by a cardiologist. At the end of the process, an anaesthesiological assessment is performed, and surgical clarification is legally needed.

To ensure altruistic donations in accordance with German law, donor operations take place after sufficient time for consideration and after a positive vote by an ethics living donation committee (anchored in the state medical association of the respective federal state).

In the case of a tumour indication for transplantation or technical considerations in the recipient, the living donation procedure starts with the exploration of the recipient first. If this is ruled out, surgical exploration of the donor is performed, including intraoperative cholangiography. Donor and recipient surgeries were performed nearly in parallel in two operating theatres.

### Analysis

For evaluation, the various processes should be broken down, and the potential donors who presented themselves as outpatients and inpatients should be evaluated. Relationships with the recipient should also be examined according to the following groups: parents/adults (grade 1), siblings/adults/grandparents (grade 2), spouses (grade 3), and distant relatives or friends (grade 4).

In addition, reasons for rejecting potential living donors should be presented and evaluated. For this purpose, we divided the reasons for rejection into seven categories, as shown in Table 3 .

**Table:** **Table 3** Exclusion Criteria.

Exclusion Criteria	
**Potential Donors Who were not accepted**	(n= 470) *
*Relative contraindications*	
*Donor-related*	
Body-mass-index > 30 kg/m² ± Steatosis hepatis ± previous abdominal operations	52 (11,1%)
Blood group incompatibility	24 (5,1%)
another donor is more suitable for LDLT	76 (16,2%)
*Recipient-related*	
deceased donor transplantation was performed	40 (8,5%)
*Absolute contraindications*	
*Donor-related*	
withdrawal of consent to donate	20 (4,3%)
Rejection for medical or mental reasons (for example: cardiac or pulmonary contraindications)	29 (6,2%)
Concomitant medication with perioperatively increased risk of bleeding	17 (3,6%)
Concomitant medical condition or medication that has an increased perioperative risk of wound healing disorders	46 (9,8%)
Lack of technical aptitude	16 (3,4%)
*Recipient-related*	
Lack of acceptance by the recipient	34 (7,2%)
disease progress in the recipient	90 (19,1%)
**Potential Donors who were accepted**	132
*Successful donation*	124
*Aborted Procedure (intraoperative cancellation because of anatomical variations or steatosis/fibrosis or circulatory instability of the recipient during the operation or progression of the underlying disease in the recipient)*	13
* Several reasons for rejection can occur simultaneously (which explains why the total number of rejections in this table is greater than the number of patients).	

During the evaluation of recipient data, three groups were distinguished depending on liver function and liver disease: patients with noncirrhotic liver disease, patients with non-decompensated liver cirrhosis, and patients with decompensated liver cirrhosis. In addition, the Model for End-Stage Liver Disease (MELD) score of the patients and exceptional points were recorded. In the case of hepatocellular carcinoma, classification according to the Milan criteria was used 24 .

To examine the outcomes of living donation performed on the donor, the results were evaluated retrospectively. The primary endpoints for donor outcomes were donor mortality and donor morbidity (based on the Clavien–Dindo classification). The secondary endpoints were biliary complications (biliary leakage, classified based on the Nagano classification), major cardiac events after living donation (MACE), postoperative small-for-size syndrome (SFSS), hospital stay, and ICU stay associated with the donation process.

The collected data were read retrospectively using electronic data processing programs and, in individual cases, supplemented by telephone queries. Owing to the retrospective, single-centre approach, a good comparison with alternative evaluation methods was not possible.

### Donor safety

Data from all 124 living liver donors were prospectively collected and documented in a dedicated transplant database. Clinical parameters such as length of hospital and ICU stay, postoperative complications (classified according to the Clavien–Dindo system), and any surgical or psychological interventions were systematically recorded during the initial hospital stay and subsequent follow-up visits. All complications and follow-up findings were verified through clinical examination, imaging, operative reports, and patient interviews. Donors are enrolled in a structured, lifelong follow-up program, with clinical evaluations recommended at least annually. The adherence rate to these regular follow-up visits exceeds 90%, enabling continuous assessment of long-term outcomes, including late-onset complications such as incisional hernias or cardiovascular events.

## Results

**Fig. 1:**
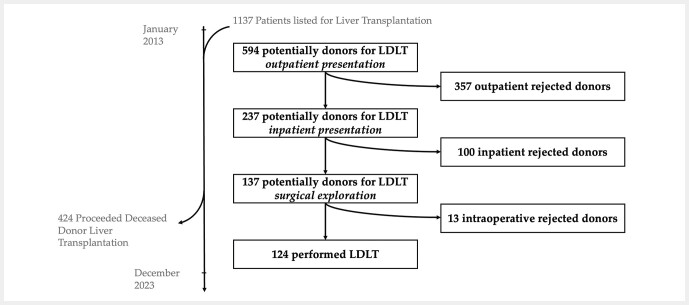
Flowchart.

Among the 320 recipients, 594 potential living liver donors were evaluated at Jena University Hospital between January 2013 and December 2023. Of these, 357 candidates were rejected after outpatient presentation, and 237 were admitted for further inpatient evaluation. Following additional examinations, 113 of these 237 candidates were excluded, resulting in 124 successful living-donor liver transplantations ( Fig. 1 ).

The demographic and relationship characteristics of all potential donors are summarized in Table 2 . The mean donor age did not differ significantly between accepted and nonaccepted candidates (43.8 ± 12.0 years vs. 43.7 ± 12.6 years; p = 0.17). There was a slight predominance of female donors among accepted candidates (58.9% vs. 50.2%; p = 0.09), although this difference did not reach statistical significance.

However, the biological relationship between the donor and recipient was significantly associated with donor acceptance. Accepted donors were more frequently biologically related (64.5% vs. 54.5%; p = 0.04) and particularly first-degree relatives (43.5% vs. 34.5%; p = 0.06), whereas nonbiologically related donors were significantly less likely to be accepted (35.5% vs. 45.5%; p = 0.03). Similarly, distant relationships (grade 4) were significantly less frequent among accepted donors (10.5% vs. 22.1%; p < 0.01). These findings suggest that a closer familial relationship plays an important role in donor selection, likely reflecting both medical and psychosocial considerations.

Recipient characteristics also differed significantly between accepted and nonaccepted donor pairs. The mean labMELD score was significantly lower in recipients of accepted donors (11.9 vs. 15.5; p < 0.01), indicating that LDLT was performed more often in patients with less advanced liver disease. No significant differences were observed regarding the underlying disease aetiology (malignant vs. benign; p = 0.82) or tumour stage (HCC within vs. outside the Milan criteria; p = 0.15 and p = 0.75, respectively).

Of the 594 potential donors, 216 were parents or adult children (grade 1); 120 were siblings, adult grandchildren, or grandparents (grade 2); 141 were spouses or partners (grade 3); and 117 were distant relatives or friends (grade 4). Table 3 provides a detailed breakdown of the reasons for donor rejection.

The underlying disease among the recipients was malignant in 60 patients and benign in 64 patients ( Table 1 ). Hepatocellular carcinoma accounted for the largest proportion of malignant cases; among 31 HCC patients, 7 met the Milan criteria, while 24 were outside the Milan criteria. The proportion of recipients with cirrhosis at the time of transplantation was 74.7% (239 of 320 recipients). Living donor liver transplantation was performed significantly more often in recipients without cirrhosis than in those with cirrhosis (49 of 125 vs. 31 of 195; p < 0.001).

### MELD Score and Waiting Time

In evaluating patients by entity, the mean labMELD score was 12.0 for patients with malignant tumour disease and 17.8 for patients with benign disease (p<0.01). Among the patients who underwent living-donor liver transplantation, 13 received exceptional MELD scores on the waiting list based on their liver disease. Ten of these patients had cancer with an average score of 23.0 points (each with hepatocellular carcinoma inside Milan). Three patients had benign underlying disease with an average score of 21.3 points (one patient with polycystic liver degeneration, one patient with primary sclerosing cholangitis, and one patient with secondary sclerosing cholangitis).

Furthermore, two patients had exceptional points on the waiting list because of a nonstandard-exceptional MELD: a patient with liver metastases from a neuroendocrine tumour of the small intestine and a patient with decompensated nutritional–toxic liver cirrhosis with recurrent bacterial peritonitis and ascites fistula via an umbilical hernia.

There was no significant difference in the Eurotransplant waiting time between patients with malignant disease and those with nonmalignant disease (235.3 d vs. 437.5 d; p=0.263). Significantly more potential donors were presented for recipients with malignant disease who were considered for living donation (2.0 vs. 1.5; p=0.023).

**Fig. 2:**
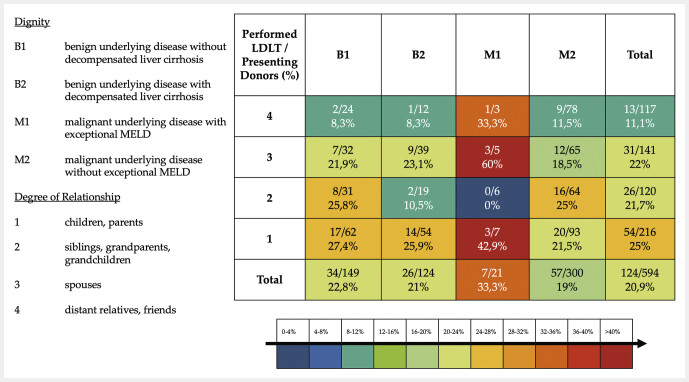
Degree of Relationship vs. Diagnosis. Color scale indicates the proportion of performed living donor liver transplantations (LDLT) among presenting donors in each category. The percentages are grouped and color-coded as follows: Dark blue 0–4%, Blue 4–8%, Teal 8–12%, Green 12–16%, Yellow-Green 16–20%, Yellow 20–24%, Orange 24–28%, Red-orange 28–32%, Bight Red 32–36%, Dark Red > 36–40%; Dark Red >40%. The terms “children” and “grandchildren” refer to the donor–recipient relationship only; all donors were adults (≥18 years).

We stratified all potential donors between 2013 and 2023 according to the underlying diagnosis of the potential recipient and the degree of relationship with the recipient and highlighted the living donations that were successfully performed ( Fig. 2 ). At our centre, we treated patients with colorectal liver metastases as part of the liver-t(w)o-Heal study 25 with two-stage living-donor liver transplantation 26 27 . Since this was not a standard procedure for all the cases, we subsequently eliminated these cases from the analysis.

### Donor rejection patterns

The main reasons for donor rejection are summarized in Table 4 . Most exclusions were related either to recipient-dependent factors – such as disease progression (n = 90) or the availability of a more suitable donor (n = 76) – or to donor-related medical contraindications (category II). Among the latter, metabolic and cardiovascular risk factors predominated, including a body mass index > 30 kg/m² (n = 20) and hepatic steatosis (n = 22). Additional frequent exclusion causes were significant comorbidities or chronic medication with increased perioperative risk, such as rheumatoid arthritis or psoriasis requiring immunosuppressive therapy (n = 17), long-standing diabetes mellitus (n = 14), and Factor-V-Leiden mutation (n = 6). Anatomical ineligibility (category III; n = 24) and blood group incompatibility (n = 24) accounted for smaller proportions.

**Table:** **Table 4** Rejection Categories.

	Definition	number of patients in our population and breakdown into different reasons for exclusion (n=)*
I	Lack of acceptance by the recipient or withdrawal of consent to donate	54 Lack of acceptance by the recipient (a donor presented himself on his own initiative willingness for donation and the recipient declined a living donation) or withdrawal of consent to donate
II	For medical or psychological reasons	**52** Body mass index > 30 kg/m² (n=20), Steatosis hepatis (n=22), liver cirrhosis or fibrosis (n=4), previous abdominal operations (especially in the upper abdomen) with significant intra-abdominal adhesions (n=6).
		**46** Concomitant medical condition or medication with an increased perioperative risk of wound healing disorders: rheumatoid arthritis (n=11) or psoriasis with methotrexate (n=4) or monoclonal antibody (n=2) or prednisolone (n=6) or azathioprine therapy (n=3). Long-standing diabetes mellitus (n=14). Factor-V-Leiden mutation (n=6).
		**22** Cardiac contraindications: coronary heart disease (n=5), chronic heart failure (n=4), conspicuous cardiac diagnostics (poor exercise capacity or ST segment changes in the exercise electrocardiogram) (n=13)
		**17** Concomitant medication with perioperatively increased risk of bleeding: dual antiplatelet agents (n=7), therapeutic anticoagulation (n=10).
		**16** Diseases diagnosed during donor evaluation: suspected malignancy (n=7) or other diseases (sarcoidosis, collagenosis, colitis, neurodegenerative disease; n=8).
		**7** Pulmonary contraindications: pulmonary disease (Chronic obstructive pulmonary disease ≥ GOLD II, n=4), conspicuous pulmonary diagnostics (spirometry shows severe ventilation disorder, n=3).
III	Lack of technical eligibility	**24** Lack of technical feasibility: preoperative diagnosed anatomical variant (n=16), intraoperative cancellation of the donation in the case of an unexpected anatomical variant with a substantial risk of donor morbidity (n=8)
IV	Another donor is more suitable for LDLT	**76**
V	Disease progress in the recipient	**90** Disease progress in the recipient (aborted during the evaluation process or after operational exploration of the recipient (extrahepatic tumour involvement in patients with an underlying malignant disease or fulminant decompensated liver function in a benign underlying disease)
VI	Blood group incompatibility (In principle, an ABO-incompatible living donation is feasible, but should be evaluated on a case-by-case basis)	**24**
VII	Deceased donor transplantation was performed	**40** deceased donor transplantations were performed
*Often, there are several reasons for rejection at the same time (which explains why the total number of rejections in this table is higher than the number of patients).		

A distinction between outpatient rejection and inpatient rejection is shown in Table 5 . Donors were most frequently excluded during the outpatient evaluation when clear contraindications were evident – particularly in cases of rheumatoid arthritis (p < 0.001), diabetes mellitus (p = 0.002), and psoriasis or obesity with metabolic syndrome. In contrast, donors were more often rejected during inpatient assessment when complex diagnostic findings emerged, such as conspicuous cardiac or pulmonary diagnostics (p < 0.001) or suspected malignancy (p = 0.015), which required advanced imaging or interdisciplinary work-up. These data demonstrate that early outpatient screening effectively identifies obvious contraindications, whereas rarer and diagnostically challenging findings are typically recognized only in the inpatient phase.

**Table:** **Table 5** Distinction between outpatient and inpatient donor rejection. P values were calculated using Fisher’s exact test (two-sided).

Disease (n)	rejected as a donor during the outpatient presentation (n)	rejected as a donor during the inpatient presentation (n)	***p* value **
Coronary heart disease (n=5)	0	5	0.062
Chronic heart failure (n=4)	2	2	1.000
Chronic obstructive pulmonary disease ≥ GOLD II (n=4)	4	0	0.124
Conspicuous cardiac or pulmonary diagnostics (n=16)	0	16	<0.001
Vascular or biliary anatomic anomaly (n=24)	16	8	0.147
Liver fibrosis/cirrhosis (n=4)	1	3	0.624
Primary sclerosing cholangitis (n=1)	0	1	1.000
Rheumatoid arthritis* (n=11)	11	0	<0.001
Psoriasis* (n=4)	4	0	0.124
Diabetes mellitus (n=14)	13	1	0.002
Factor-V-Leiden mutation (n=3)	3	0	0.249
Suspected malignancy (n=7)	0	7	0.015
Sarcoidosis (n=5)	4	1	0.374
Collagenosis (n=1)	0	1	1.000
Colitis (n=1)	1	0	1.000
Neurodegenerative Disease (n=1)	1	0	1.000
*Rejection due to disease-specific immunomodulatory medications			

Table 6 differentiates between rejection due to preexisting conditions and diseases newly diagnosed during donor evaluation. Most metabolic and autoimmune disorders – such as diabetes mellitus (p < 0.001) and rheumatoid arthritis (p < 0.001) – were already known before the evaluation process and were thus excluded early. In contrast, vascular or biliary anatomical anomalies (p < 0.001) and newly detected cardiac abnormalities (p < 0.001) were typically discovered only during detailed imaging or diagnostic work-up. These findings emphasize the importance of thorough preoperative imaging and cardiopulmonary assessment to identify previously undetected contraindications.

**Table:** **Table 6** Distinction between donor rejection for preexisting conditions or incidental diseases. P values were calculated using Fisher’s exact test (two-sided).

Disease (n=total)	Rejection as a donor due to a known preexisting condition/disease (n)	Rejection as a donor due to a condition that was diagnosed during the evaluation (n)	*p* value
Coronary heart disease (n=5)	3	2	1.000
Chronic heart failure (n=4)	2	2	1.000
Chronic obstructive pulmonary disease ≥ GOLD II (n=4)	4	0	0.124
Conspicuous cardiac or pulmonary diagnostics (n=16)	0	16	<0.001
Vascular or biliary anatomic anomaly (n=24)	0	24	<0.001
Liver fibrosis/cirrhosis (n=4)	1	3	0.624
Primary sclerosing cholangitis (n=1)	0	1	1.000
Rheumatoid arthritis (n=11)	11	0	<0.001
Psoriasis (n=4)	4	0	0.124
Diabetes mellitus (n=14)	14	0	<0.001
Factor-V-Leiden mutation (n=3)	3	0	0.249
Suspected malignancy (n=7)	0	7	0.015
Sarcoidosis (n=5)	0	5	0.062
Collagenosis (n=1)	0	1	1.000
Colitis (n=1)	0	1	1.000
Neurodegenerative Disease (n=1)	1	0	1.000

Taken together, these findings suggest that donor exclusion most frequently results from metabolic, anatomical, or cardiopulmonary conditions. The combination of structured outpatient screening and comprehensive inpatient evaluation enables stepwise and safe donor selection while minimizing unnecessary risk exposure.

### Donor Outcome

The 124 donors who received living donations had an average hospital stay of 13.9 days (range 5–91) and an ICU stay of 1.8 days (range 0–31). The patient with a postoperative hospital stay of 91 days developed postoperative small-for-size syndrome (SFSS) with a prolonged recovery period. The patient has since made a full recovery and is currently without any residual limitations. The overall morbidity and mortality rates were 23.6% and 0%, respectively. Surgical complications were graded according to the Clavien–Dindo classification, as detailed in Table 7 . The most common complication was biliary leakage, which was treated with endoscopic interventions (stenting and papillotomy) in 14 donors with or without percutaneous drainage of biliomas. Four patients required early reoperation (entrapped surgical drainage, suspected biliary peritonitis, abdominal wound dehiscence, and surgical site haematoma). One donor had transient small-for-size syndrome. One patient experienced a major cardiac event 14 months after donation, revealing coronary stenting. Eighteen incisional hernias were operated upon during long-term follow-up. Eleven donors received psychological care because of psychological distress related to the donation and its consequences.

**Table:** **Table 7** Donor morbidity depends on the donated liver segment (right hemihepatectomy, left hemihepatectomy, or left lateral resection of segments II and III). Categorical variables were compared using Fisher’s exact test (two-sided), and continuous variables were compared using the Kruskal–Wallis test.

	Right-lobe (n=101)	Left-lobe (n=11)	Left-lateral-lobe (n=11)	*p value*
Complications* (n [%])	24 (23,8%)	3 (27,3%)	2 (18,2%)	0.92
Biliary Leakage** (n [%])	20 (19.8%)	2 (18.2%)	2 (18.2%)	1.00
Reoperation (n [%])	3 (3%)	1 (9.1%)	0 (0%)	0.55
Incisional hernia (n [%])	14 (13.9%)	4 (36.4%)	0 (0%)	0.05
ICU-Stay (mean ± SD)	1.9 ± 3	1.3 ± 0.5	1.1 ± 0.5	0.51
Hospital-Stay (mean ± SD)	14.5 ± 10.2	12.9 ± 9.6	9.7 ± 7.1	0.31
Rehospitalization (n [%])	14 (13.9%)	1 (9.1%)	1 (9.1%)	1.00
* – All complications classified as Clavien–Dindo grade ≥ 3a ** – Bile leaks of all severity grades (regardless of the need for treatment) and classified according to the Nagano system as follows: Nagano A (n = 18), Nagano B (n = 5), Nagano C (n = 1), and Nagano D (n = 0). Biliary Complications were further graded using the Clavien–Dindo classification: grade II (n = 14), grade IIIa (n = 8), and grade IIIb (n = 2). Reasons for reoperations included: bile leakage classified as Nagano C; hemaskos due to secondary haemorrhage; and burst abdomen caused by fascial dehiscence without intra-abdominal pathology				

## Discussion

This study aimed to describe a structured evaluation pathway for living liver donors, using real-world data to highlight key decision points, common contraindications, and logistical improvements.

The standardized protocol applied at our centre emphasizes early outpatient evaluation, often remote or minimally invasive, to reduce hospitalization requirements while ensuring safety.

Particular focus is placed on early identification of absolute and relative contraindications to avoid unnecessary investigations. Our experience confirms that a notable proportion of seemingly healthy donors present with previously undetected medical conditions requiring exclusion from donation.

We observed that the willingness to donate among potential donors varies significantly depending on the recipient’s underlying diagnosis and urgency. In cases of malignant disease or decompensated liver cirrhosis, the donor pool often expands to include more distant relatives or friends, reflecting the moral urgency perceived by donors. This behavioural response aligns with our finding that patients with higher urgency diagnoses were more likely to attract multiple donor candidates.

Structured and staged outpatient evaluation contributes to process efficiency and resource stewardship, as reported previously 6 28 29 .

Living donation represents an important therapeutic alternative, particularly in the setting of decompensated disease with low MELD or malignancy, without exception of MELD eligibility 30 31 32 33 34 35 .

Our data illustrate that for patients with cancer, MELD scoring often underrepresents clinical urgency. These patients may be excluded from the waiting list because of disease progression before organ allocation 30 36 37 38 39 40 .

LDLT offers a viable pathway for these patients and has been associated with a broader willingness to donate among extended social circles.

While the increase in donor interest for oncological recipients was statistically significant, a similar but nonsignificant trend was observed for patients with decompensated cirrhosis. Another major advantage of LDLT is the ability to plan the intervention, which reduces the overall waiting time compared with that of DDLT.

Based on our experience, exclusion criteria for donation, including both absolute and relative contraindications, were clearly defined ( Table 4 ). Outpatient presentation allowed early exclusion in many cases ( Table 5 ), and we frequently uncovered significant, previously unknown conditions in donors thought to be healthy ( Table 6 ). Absolute contraindications, such as cardiac comorbidity, must be strictly observed because of the elevated perioperative risk. Relative contraindications, such as ABO incompatibility, are assessed individually, depending on clinical alternatives 41 .

We developed a standardized model for donor evaluation that is now reflected in the newly published German S2k guideline on liver transplantation. The chapter on living donation was developed in accordance with our protocol and formally incorporates these evidence-based strategies 42 .

Initial contraindications can often be detected in outpatient settings via clinical history and basic diagnostics, sparing donors from invasive testing and unnecessary hospitalization.

Donor evaluation may be initiated by the donor themselves, with final feasibility dependent on recipient consent and interdisciplinary risk-benefit assessment. Specific attention is given to early cardiopulmonary risk stratification and a detailed family history of coagulation disorders.

Given the repeated detection of incidental pathologies — including malignancy — interdisciplinary review of imaging and laboratory findings is essential.

All the imaging data should be reviewed by experienced radiologists and discussed with the transplant surgeon, especially those concerning vascular or biliary anomalies. In our practice, 3D reconstruction and volumetry are routinely used to select the optimal surgical approach (right vs. left split).

Psychological support is initiated early and maintained throughout evaluation, donation, and follow-up to ensure continuity of care 43 44 . This support is also extended when donation does not proceed to protect relatives from distress, guilt, or coercion 45 46 .

The principle of subsidiarity was actively applied in this cohort. Patients were evaluated for LDLT while remaining on the Eurotransplant waiting list. Between 2013 and 2023, 320 patients were evaluated for LDLT. Of these, 124 underwent living-donor transplantation. Of the remaining 196 patients, 135 ultimately received DDLT. This dual-pathway approach ensures timely transplantation, especially in high-risk groups without MELD exceptions. Patients with HCC outside the Milan criteria, those without cirrhosis, or those included in clinical trials (e.g., Liver-t(w)o-heal) were especially likely to undergo LDLT.

Subsidiarity also manifested through centre-specific policies adapted to local organ availability, infrastructure, and collaboration with other centres. Donors were sometimes excluded because of the accumulation of multiple minor contraindications rather than a single definitive contraindication.

Psychiatric concerns are often accompanied by physical findings to support ethical decision-making 46 47 . We emphasize that no donors were excluded solely based on psychological criteria.

Donor evaluation can also reveal previously unknown diseases in otherwise healthy candidates, as has also been shown in other programs 48 .

To enhance screening, we have added colonoscopy for donors aged over 50 years, gynaecological exams for women, and urological exams for men. While data supporting these additions are limited, they reflect increasing donor age and evolving risk profiles.

Liver biopsy remains the gold standard for assessing steatosis and parenchymal changes 17 , but its use remains selective. Noninvasive imaging, including MRI and contrast-enhanced CT, can detect severe steatosis or fibrosis but lacks precision for detecting intermediate disease.

In our program, donors with unclear imaging findings are more likely to be excluded than those subjected to biopsy. Particularly in cases of borderline hepatic steatosis, we refrain from performing liver biopsy, as the procedure has its own risk of complications, and the probability that histological results would still allow for safe donation is very low. Considering the high sensitivity and specificity of current imaging modalities for detecting clinically relevant steatosis, this conservative strategy appears justified from a risk–benefit perspective.

### Donor Outcome

Donor safety remains a fundamental concern in living-donor liver transplantation (LDLT). In our cohort, the donor safety data are consistent with reports from other high-volume centres ( Table 7 ). The overall morbidity rate was 23.6%, with biliary leakage being the most frequent complication (11.3%), which is consistent with published data 49 50 51 52 . Most biliary complications are successfully managed by endoscopic treatment, including stenting or papillotomy. The incidence of incisional hernia was 14.5%, which is slightly higher than the average but still within the published range (5–13%) 53 54 . Severe complications (Clavien–Dindo ≥ IIIb) were rare (3.2%), and no donor deaths occurred in our series. Lifelong follow-up, with 90% adherence, enables early identification and management of late complications, including cardiovascular or abdominal wall problems.

Psychological distress after living liver donation was observed in several donors, and while postoperative complications — particularly prolonged courses related to biliary leakage — can contribute to this burden, our experience suggests that donor distress often arises independently of donor morbidity 55 . In particular, emotionally challenging recipient outcomes, such as early postoperative mortality, vascular complications, graft nonfunction, or recurrence of malignant disease, appear to play a substantial role in shaping donors’ postdonation psychological trajectories. These findings highlight the importance of comprehensive preventive strategies, including thorough predonation counselling that realistically addresses potential unfavourable recipient outcomes, continuous psychological support throughout the entire perioperative course, and close, transparent communication within a trusted physician–donor relationship to mitigate the risk of long-term distress 43 45 46 .

Although our centre performs a relatively high number of LDLTs according to European standards, the overall case volume remains modest compared with that of major Asian centres, where LDLT is the predominant form of liver transplantation. Nevertheless, our results demonstrate that donor outcomes remain comparable to international benchmarks, as evidenced by the large meta-analysis by Xiao et al. involving 60,829 living liver donors. The study reported an overall complication rate of approximately 25%, a major complication rate of 5.5%, and a donor mortality rate of 0.06% 55 . These data confirm that our donor morbidity and safety outcomes are within the internationally expected range, even in a lower-volume Western setting.

However, interpretation of international outcome data requires caution. As emphasized by Ringe and Strong, a potential publication bias may exist, since donor mortality and severe adverse events are not consistently reported across centres 56 . This underreporting could contribute to an overly optimistic representation of LDLT safety. Furthermore, Mulligan has already highlighted the lack of a centralized, mandatory global registry for living liver donors as a major limitation in accurately assessing and comparing donor risk 57 . The absence of such a reporting system hinders transparency, impedes long-term outcome monitoring, and makes it difficult to capture rare but serious donor complications.

Taken together, our findings underscore that LDLT can be performed safely and ethically in experienced centres with structured donor evaluation, meticulous perioperative management, and long-term follow-up protocols 49 50 58 . Although donor morbidity is not negligible, complications are generally manageable and rarely life-threatening. Nonetheless, given the very low but nonzero risk of mortality (0.1–0.3%) 59 , ongoing efforts towards comprehensive international data collection, transparent reporting, and sustained psychological and medical donor support are essential to ensure continued safety and public trust in LDLT programs.

### Limitations

This study focused on adult-to-adult LDLT. Differences in paediatric protocols (e.g., biopsy practices and donor profiles) limit generalizability 60 .

## Conclusion

In conclusion, this study provides a comprehensive analysis of the entire living donor evaluation process within a European transplant program. While large-scale studies on donor evaluation have thus far originated almost exclusively from Asian or North American centres, this work offers the first systematic insight into the European context, where the framework and ethical conditions of living donation differ substantially. By statistically identifying key reasons for donor rejection and demonstrating the safety of a structured stepwise evaluation process, our findings highlight the feasibility of expanding living-donor liver transplantation in Europe and contribute essential evidence for optimizing donor selection and safety standards.

In our opinion, living liver donation programs for adult patients can also be expanded to Europe, and further concepts for diseases not previously accessible via deceased-donor liver transplantation, such as colorectal liver metastases or perihilar cholangiocarcinoma, can be established.

### Institutional Review Board Statement

The study was conducted in accordance with the Declaration of Helsinki and approved by the ethics committee of the University Hospital Jena on March 13, 2024 (2024–3266-Data).

### Informed Consent Statement

Informed consent was obtained from all the subjects involved in the study.

### Data Availability Statement

The data presented in this study are available upon request from the corresponding author.
